# Thyrotroph Pituitary Neuroendocrine Tumors: Molecular Pathology, Diagnostic Challenges, and Receptor-Targeted Therapeutic Strategies

**DOI:** 10.3390/cancers18050838

**Published:** 2026-03-04

**Authors:** Kazunori Kageyama, Keisuke Sato, Mizuki Tasso, Yuki Nakada

**Affiliations:** Division of Diabetes, Metabolism and Endocrinology, Faculty of Medicine, Tohoku Medical and Pharmaceutical University, 1-15-1 Fukumuro, Sendai 983-8536, Japan

**Keywords:** TSHoma, thyrotroph, pituitary neuroendocrine tumor, hyperthyroidism, PIT1, somatostatin receptor

## Abstract

Thyrotroph pituitary neuroendocrine tumors (PitNETs) are rare functional pituitary tumors characterized by autonomous secretion of thyroid-stimulating hormone (TSH), leading to central hyperthyroidism. Under the 2022 World Health Organization classification, these tumors are defined as PIT1-lineage PitNETs, reflecting lineage-specific differentiation and improving pathological accuracy. Clinically, thyrotroph PitNETs often present as macroadenomas with invasive growth, making complete surgical resection challenging and necessitating multimodal treatment strategies. From a molecular oncology perspective, thyrotroph PitNETs lack recurrent driver mutations and instead exhibit heterogeneous alterations involving dysregulated cell-cycle control, impaired thyroid hormone-mediated negative feedback, and aberrant growth factor signaling. Immunohistochemically, tumor cells express PIT1 and TSH and show strong membranous expression of somatostatin receptor subtype 2, providing a biological rationale for somatostatin receptor ligand -based therapy. Somatostatin receptor ligands play a central role in the management of thyrotroph PitNETs as preoperative, adjuvant, or primary treatment and achieve effective hormonal control and tumor stabilization or shrinkage in many patients. Accurate differentiation between thyrotroph PitNETs and resistance to thyroid hormone β is essential, as these entities share biochemical features but require fundamentally different management. Advances in lineage-based tumor classification, receptor profiling, and molecular pathology have refined diagnostic strategies and enabled a more personalized, tumor-oriented therapeutic approach. This review highlights current insights into the tumor biology and treatment of thyrotroph PitNETs and discusses future perspectives for receptor-targeted and molecularly informed therapies.

## Simple Summary

Thyrotroph pituitary neuroendocrine tumors (PitNETs), also known as thyroid-stimulating hormone (TSH)-producing pituitary adenomas (TSHomas), are rare functional pituitary tumors that cause excessive thyroid hormone production. These tumors are often difficult to cure by surgery alone, because they are frequently large or invasive at diagnosis. A key biological feature of thyrotroph PitNETs is their high expression of somatostatin receptor subtype 2, which makes them particularly sensitive to somatostatin receptor ligand therapy. Recent advances in tumor classification and molecular pathology have improved our understanding of their tumor biology and therapeutic vulnerabilities. This review summarizes current knowledge of the molecular mechanisms, pathological features, diagnostic challenges, and treatment strategies for thyrotroph PitNETs, with a focus on receptor-targeted therapies that bridge tumor biology and clinical management.

## 1. Introduction

Thyrotroph pituitary neuroendocrine tumors (PitNETs), historically referred to as thyroid-stimulating hormone (TSH)-producing pituitary adenomas (TSHomas), are rare functional pituitary tumors characterized by autonomous secretion of TSH despite elevated circulating thyroid hormone levels. According to the 2022 World Health Organization (WHO) Classification (2022 WHO classification) of Endocrine and Neuroendocrine Tumours (5th edition) [1], these neoplasms are classified as thyrotroph PitNETs, defined by lineage-specific differentiation and PIT1 expression. Clinically, thyrotroph PitNETs cause inappropriate TSH secretion, leading to central hyperthyroidism with variable clinical manifestations, ranging from overt thyrotoxicosis to relatively mild or atypical symptoms.

Excessive and autonomous TSH secretion by thyrotroph PitNETs results in persistent stimulation of the thyroid gland and increased production of triiodothyronine (T3) and thyroxine (T4). Consequently, patients may develop significant systemic complications, including atrial fibrillation, heart failure, osteoporosis, weight loss, neuropsychiatric disturbances, and impaired glucose metabolism [2]. Transsphenoidal surgical resection remains the first-line treatment for thyrotroph PitNETs. However, complete surgical remission is often difficult to achieve because these tumors are frequently large at diagnosis, invasive, and fibrotic. Therefore, adjuvant medical therapies are commonly required to control both hormonal hypersecretion and tumor progression.

A distinctive feature of thyrotroph PitNETs is their high sensitivity to somatostatin receptor ligands (SRLs), which reflects the abundant expression of somatostatin receptor subtype 2 (SSTR2) and, to a lesser extent, somatostatin receptor subtype 5 (SSTR5) on tumor cells [3]. Immunohistochemical and functional studies have consistently demonstrated that SSTR2 is the predominant somatostatin receptor subtype in thyrotroph PitNETs, mediating potent inhibitory effects on TSH secretion [4,5]. Clinically, long-acting SRLs, such as octreotide and lanreotide, effectively suppress TSH secretion, normalize thyroid hormone levels, and induce tumor shrinkage in a substantial proportion of patients. Accordingly, SRLs play a central role not only as adjuvant therapy following incomplete surgery but also as preoperative treatment to achieve euthyroidism and reduce surgical risk, and in selected cases, as primary therapy.

From a molecular and pathophysiological perspective, thyrotroph PitNETs exhibit dysregulation of thyroid hormone-mediated negative feedback. Compared with normal thyrotrophs, tumor cells demonstrate reduced sensitivity to circulating thyroid hormones, requiring supraphysiological levels of T3 and T4 to suppress TSH secretion [6]. Proposed mechanisms include altered expression or function of thyroid hormone receptor β (TRβ), impaired recruitment of transcriptional corepressors, and abnormalities in chromatin remodeling complexes involved in negative feedback regulation of the *TSHβ* gene [7]. These alterations contribute to autonomous TSH production and sustained tumor activity.

In contrast to corticotroph PitNETs, recurrent somatic mutations such as those affecting the *ubiquitin-specific protease 8* (*USP8*) gene are uncommon in thyrotroph PitNETs [8]. Instead, tumorigenesis appears to be driven by a combination of aberrant growth factor signaling, dysregulated cell-cycle control, and epigenetic modifications [9]. Increased expression of cell-cycle regulators, including cyclins, together with reduced expression of cell-cycle inhibitors such as p27Kip1, has been reported [10], suggesting that disruption of cell-cycle regulation contributes to tumor proliferation. Elucidation of these molecular mechanisms is essential for the development of targeted therapies beyond conventional SSAs and for refining individualized treatment strategies for patients with thyrotroph PitNETs. Differential diagnosis of inappropriate TSH secretion is discussed in detail in Section 5.

This narrative review was based on a literature search performed in PubMed and Embase up to November 2025 using combinations of the terms “thyrotroph PitNET,” “TSH-producing adenoma,” “TSHoma,” “somatostatin receptor,” “RTHβ,” and “central hyperthyroidism.” Priority was given to original clinical series, translational studies, and publications within the last five years where available.

## 2. Classification and Epidemiology of Thyrotroph PitNET

### 2.1. Classifications

Thyrotroph PitNETs, historically referred to as TSH-producing pituitary adenomas, are rare functional pituitary tumors characterized by autonomous secretion of TSH. In the 2022 WHO classification, these tumors are categorized as thyrotroph PitNETs, defined by lineage-specific differentiation within the PIT1 lineage, together with immunohistochemical expression of TSH [1]. This lineage-based classification replaces the traditional adenoma-based terminology and reflects a conceptual shift toward recognizing pituitary tumors as neuroendocrine neoplasms with distinct biological behavior [11].

### 2.2. Epidemiology

Recent cohort data indicate an incidence of approximately 0.15–0.26 per million population per year and a prevalence of 1–3 per million, with diagnosis frequently occurring in middle-aged adults and significant diagnostic delay documented in modern series [12]. Epidemiologically, thyrotroph PitNETs account for approximately 0.5–2% of all PitNETs and represent one of the rarest functional PitNET subtypes. They are typically diagnosed in adulthood and often present as macrotumors at the time of diagnosis, reflecting delayed recognition due to subtle or atypical clinical manifestations of central hyperthyroidism. At diagnosis, 70–80% of patients present with macrotumors, and cavernous sinus invasion is reported in approximately 30–40% of cases. No clear sex predominance has been consistently demonstrated. Diagnostic delay is common and often exceeds several years. Contributing factors include biochemical misinterpretation, inappropriate treatment with antithyroid drugs, and failure to recognize the pattern of inappropriate TSH secretion. Increased awareness of diagnostic pitfalls is essential to reduce delayed diagnosis and tumor progression.

Although most cases are sporadic, rare associations with familial pituitary tumor syndromes, such as multiple endocrine neoplasia type 1 (MEN1) and familial isolated pituitary adenoma, have been reported [13,14]. The rarity and clinical heterogeneity of thyrotroph PitNETs underscore the importance of accurate classification, early recognition, and a multidisciplinary approach to diagnosis and management. This classification provides a biological framework linking tumor lineage to hormone secretion and therapeutic responsiveness.

### 2.3. Clinical Presentation and Diagnostic Challenges

Thyrotroph PitNETs most commonly present with manifestations of central hyperthyroidism. Symptoms of thyrotoxicosis may include palpitations, heat intolerance, weight loss, tremor, anxiety, and atrial fibrillation. However, the clinical phenotype is often milder than that observed in primary hyperthyroidism, and overt symptoms may be absent in a substantial proportion of patients. In addition to systemic manifestations of thyroid hormone excess, mass effects related to macrotumors are frequent, including headache, visual field defects, and hypopituitarism.

Several characteristics contribute to frequent diagnostic delay and misclassification. The rarity of thyrotroph PitNETs, the moderate biochemical elevation of thyroid hormones, and overlapping laboratory profiles with resistance to thyroid hormone β (RTHβ) or assay interference often lead to initial misinterpretation. Moreover, some patients are inappropriately treated with antithyroid drugs before recognition of inappropriate TSH secretion, which may further obscure the diagnosis. Awareness of these clinical and biochemical features is essential to avoid delayed diagnosis and unnecessary treatment.

## 3. Molecular Pathology of Thyrotroph PitNETs

### 3.1. Gene Mutations

Unlike corticotroph PitNETs, in which recurrent somatic mutations such as those affecting *USP8* are frequently identified, thyrotroph PitNETs lack a well-defined, recurrent driver mutation. To date, comprehensive genomic analyses have revealed a relatively low mutational burden in these tumors [15], suggesting that thyrotroph tumorigenesis is driven by heterogeneous molecular alterations rather than a single dominant genetic event. Sporadic somatic mutations affecting signaling pathways involved in cell proliferation and hormone regulation have been reported [16], but their prevalence remains low and inconsistent across studies. This genetic heterogeneity underscores the importance of epigenetic regulation and transcriptional dysregulation in thyrotroph PitNETs pathogenesis.

### 3.2. Cell-Cycle Regulators

Dysregulation of cell-cycle control represents a key mechanism contributing to tumor growth in thyrotroph PitNETs. Increased expressions of positive cell-cycle regulators, including cyclin D1 and cyclin E, have been observed in TSH-producing tumors [17]. In parallel, reduced expression of cell-cycle inhibitors, particularly p27Kip1, has been reported [18]. Loss of p27Kip1 is associated with increased proliferative activity and has been implicated in aggressive behavior in various PitNET subtypes [19]. These findings indicate that an imbalance between cell-cycle-promoting and inhibitory factors facilitates uncontrolled proliferation of neoplastic thyrotroph cells and may contribute to tumor invasiveness and resistance to complete surgical resection.

### 3.3. Genetic and Familial Syndromes

Thyrotroph PitNETs are typically sporadic; however, rare cases have been reported in the context of genetic predisposition syndromes [20]. Germline mutations associated with familial isolated pituitary adenoma, particularly those involving the aryl hydrocarbon receptor-interacting protein (*AIP*) gene, have occasionally been identified in patients with thyrotroph PitNETs [13], although their frequency is markedly lower than in somatotroph or lactotroph PitNETs. In addition, thyrotroph PitNETs have been rarely described in patients with MEN1, caused by germline mutations in the *MEN1* tumor suppressor gene [14]. These observations suggest that while familial syndromes are uncommon in thyrotroph PitNETs, genetic susceptibility may contribute to tumor development in a small subset of patients.

### 3.4. Oncogenes and Tumor Suppressor Genes

Alterations in oncogenes and tumor suppressor genes involved in pituitary cell proliferation and differentiation have been implicated in thyrotroph PitNETs [9]. Overactivation of growth factor signaling pathways, including epidermal growth factor receptor and fibroblast growth factor receptor signaling, has been associated with enhanced TSH secretion and tumor growth in some cases [21]. Conversely, reduced expression or functional impairment of tumor suppressor proteins such as p27Kip1 and MENIN may facilitate neoplastic transformation and progression [22]. Although no single oncogenic pathway appears to dominate in thyrotroph PitNETs, the cumulative effects of dysregulated proliferative signaling and loss of cell-cycle control contribute to tumorigenesis.

### 3.5. Immunohistochemical Features: PIT1, TSH, and SSTR2

Immunohistochemistry (IHC) plays a pivotal role in the diagnosis and classification of thyrotroph PitNETs under the 2022 WHO framework. These tumors are defined by expression of the lineage-specific transcription factor PIT1, confirming thyrotroph differentiation. Tumor cells demonstrate cytoplasmic immunoreactivity for TSH, which establishes the functional nature of the neoplasm. Importantly, thyrotroph PitNETs characteristically exhibit strong membranous expression of SSTR2, with variable expression of SSTR5 [3]. High SSTR2 expression correlates with clinical responsiveness to somatostatin analog therapy [4] and provides a molecular basis for the marked efficacy of SSAs in suppressing TSH secretion and reducing tumor volume. Therefore, combined evaluation of PIT1, TSH, and SSTR2 by IHC is essential not only for accurate pathological diagnosis but also for therapeutic decision-making.

### 3.6. Tumor Behavior and Proliferation Maekers

In addition to lineage markers (PIT1, TSH, SSTR2), routine pathology reporting should include proliferation markers such as Ki-67 labeling index, mitotic count, and p53 immunoreactivity. Although thyrotroph PitNETs are generally indolent, invasive macroadenomas may exhibit elevated proliferative indices.

Current clinicopathological frameworks for PitNET behavior integrate tumor size, invasiveness, and proliferative markers. While aggressive behavior is uncommon in thyrotroph PitNETs, systematic reporting facilitates risk stratification and long-term management.

## 4. Pathophysiology of Autonomous TSH Secretion

Autonomous secretion of TSH is the defining pathophysiological feature of thyrotroph PitNETs. Under physiological conditions, TSH synthesis and secretion are tightly regulated by hypothalamic TSH-releasing hormone (TRH) and negative feedback mediated by circulating thyroid hormones through TRβ. In thyrotroph PitNETs, this regulatory network is disrupted, resulting in inappropriate TSH secretion despite elevated levels of T3 and T4 [6].

### 4.1. Dysregulation of TSH Gene Transcription

At the molecular level, autonomous TSH secretion is primarily driven by dysregulation of *TSHβ* gene transcription. Thyrotroph PitNETs retain expression of the lineage-specific transcription factor PIT1, which is essential for thyrotroph differentiation and basal *TSH* gene expression. However, tumor cells exhibit altered transcriptional control, leading to constitutive activation of the *TSHβ* promoter. This dysregulation is thought to reflect an imbalance between transcriptional activators and repressors at the *TSHβ* gene locus [23], resulting in sustained hormone production independent of physiological cues.

### 4.2. Impaired Thyroid Hormone-Mediated Negative Feedback

A hallmark feature of thyrotroph PitNETs is reduced sensitivity to thyroid hormone-mediated negative feedback [24]. Compared with normal thyrotrophs, tumor cells require supraphysiological concentrations of T3 and T4 to suppress TSH secretion. Several mechanisms have been proposed to explain this partial resistance [25,26]. Altered expression or function of TRβ may impair ligand-dependent repression of *TSHβ* transcription. In addition, defective recruitment of transcriptional corepressors and chromatin remodeling complexes to the *TSHβ* promoter may further weaken negative feedback signaling. These abnormalities collectively disrupt the inhibitory effects of thyroid hormones, allowing continued TSH secretion in the presence of biochemical thyrotoxicosis. Unlike RTHβ, this impaired feedback reflects tumor-specific alterations rather than systemic hormone resistance [27].

### 4.3. Role of Hypothalamic and Paracrine Signaling

Although thyrotroph PitNETs exhibit autonomous hormone secretion, hypothalamic and local paracrine signals may still modulate tumor activity [28]. TRH receptors are often retained in tumor cells, and TRH stimulation can elicit variable TSH responses, reflecting residual responsiveness to hypothalamic input. Moreover, locally produced growth factors and cytokines within the tumor microenvironment may enhance TSH synthesis and secretion through autocrine or paracrine mechanisms [29]. The relative contribution of these pathways likely varies among tumors and may account for heterogeneity in clinical presentation and treatment response.

### 4.4. Integration with Tumor Growth and Proliferation

Autonomous TSH secretion is closely linked to tumor growth and proliferation. Dysregulated transcriptional control of hormone genes often coexists with alterations in cell-cycle regulation, including increased expressions of cyclin D1 and cyclin E and reduced expression of the cell-cycle inhibitor p27Kip1 [30]. These changes promote uncontrolled cell proliferation and may reinforce autonomous hormone production by expanding the population of hormone-secreting cells. Thus, hormonal hypersecretion and tumor growth are interdependent processes driven by shared molecular abnormalities.

### 4.5. Therapeutic Implications

Understanding the pathophysiology of autonomous TSH secretion has direct therapeutic implications. High expression of SSTR2 on tumor cells enables effective suppression of TSH secretion by somatostatin analogs, which act by inhibiting adenylate cyclase activity and downstream secretory pathways. Importantly, SRL therapy targets the tumor itself rather than peripheral thyroid hormone synthesis, distinguishing it from antithyroid drugs and reinforcing its role as disease-directed treatment. Elucidation of the molecular mechanisms underlying autonomous TSH secretion may further identify novel targets for therapy, particularly in tumors refractory to standard treatment.

## 5. Differential Diagnosis of Inappropriate TSH Secretion

Inappropriate TSH secretion, defined by elevated circulating thyroid hormone levels with non-suppressed or elevated serum TSH concentrations, requires careful differential diagnosis because it encompasses conditions with fundamentally different pathophysiological mechanisms and management strategies [31]. The major diagnostic considerations include thyrotroph PitNETs, RTHβ, assay interference, and drug-related effects. The principal diagnostic challenge is distinguishing thyrotroph PitNETs from RTHβ, while carefully excluding laboratory artifacts and other non-neoplastic causes (Table 1). Thyrotroph PitNETs are frequently misdiagnosed due to biochemical and clinical heterogeneity, reinforcing the need for standardized diagnostic approaches [32]. Differentiation between thyrotroph PitNETs and RTHβ is of paramount clinical importance, as misclassification may lead to inappropriate pituitary surgery or ineffective medical treatment.

Dynamic endocrine testing, pituitary magnetic resonance imaging (MRI), genetic analysis, and evaluation of therapeutic responsiveness should be integrated to establish an accurate diagnosis. No single test is definitive, and each modality has inherent limitations that must be recognized in clinical practice.

### 5.1. Differential Diagnosis: Thyrotroph PitNETs Versus RTHβ

A critical differential diagnosis of thyrotroph PitNETs is RTHβ, a rare hereditary disorder caused by germline mutations in the *TRβ* gene. Both conditions present with elevated circulating thyroid hormone levels in the presence of non-suppressed or inappropriately normal TSH concentrations, making biochemical differentiation challenging. However, their underlying pathophysiology is fundamentally distinct. RTHβ is characterized by systemic or tissue-selective insensitivity to thyroid hormone without autonomous TSH secretion [33], whereas thyrotroph PitNETs represent true hormone-producing neoplasms with autonomous TSH hypersecretion. Contemporary series highlight that two-thirds of patients with thyrotroph PitNETs exhibit clinical hyperthyroidism, often moderate in severity, despite markedly elevated thyroid hormone levels [34].

Several clinical, biochemical, and imaging features aid in distinguishing these two entities (Table 1). Patients with RTHβ often exhibit a family history of thyroid hormone resistance and lack progressive pituitary mass lesions [33], while thyrotroph PitNETs typically demonstrate a discrete pituitary tumor on MRI. Dynamic endocrine testing may provide additional clues, as TSH responses to TRH stimulation are frequently preserved or exaggerated in RTHβ but blunted in thyrotroph PitNETs [35]. Furthermore, serum markers reflecting peripheral thyroid hormone action, such as sex hormone–binding globulin (SHBG), tend to be elevated in thyrotroph PitNETs but remain normal or only mildly increased in RTHβ [36].

Importantly, therapeutic response to SRLs represents a key discriminator. Thyrotroph PitNETs generally express high levels of SSTR2 and respond robustly to SRLs with suppression of TSH secretion and reduction in thyroid hormone levels, whereas RTHβ shows little or no response to SRLs. Genetic analysis confirming pathogenic *TRβ* variants establishes the diagnosis of RTHβ and effectively excludes thyrotroph PitNETs [37]. Accurate differentiation between these conditions is essential, as management strategies differ substantially, with surgery and SRL therapy indicated for thyrotroph PitNETs but not for RTHβ.

### 5.2. Stepwise Diagnostic Approach and Common Pitfalls

**Figure 1:**
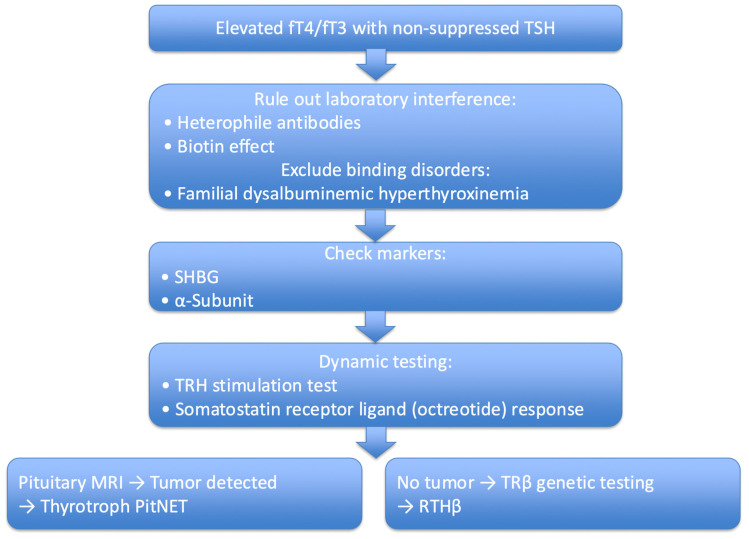
Stepwise diagnostic algorithm for inappropriate TSH secretion. The evaluation begins with confirmation of elevated fT4/fT3 levels in the presence of non-suppressed TSH. Laboratory interference and thyroid hormone binding abnormalities should be excluded before proceeding to dynamic testing (TRH stimulation test and somatostatin receptor ligand response), MRI, and genetic analysis. Somatostatin receptor ligand response may provide supportive evidence but is not diagnostic in isolation. Measurement of the glycoprotein hormone α-subunit or sex hormone–binding globulin (SHBG) may provide supportive evidence. TRβ, thyroid hormone receptor β; RTHβ, resistance to thyroid hormone β.

In clinical practice, evaluation should follow a structured and sequential pathway to avoid frequent misclassification. The initial step is confirmation of the biochemical pattern using reliable assays. Once inappropriate TSH secretion is identified, laboratory interference must be actively excluded before proceeding to imaging or dynamic testing (Figure 1).

#### 5.2.1. Exclusion of Assay Interference

Assay-related artifacts are among the most common causes of discordant thyroid function tests. Heterophile antibodies, anti-animal antibodies, and high-dose biotin supplementation may falsely elevate or alter measured thyroid hormone or TSH concentrations [38]. Repeating measurements using an alternative analytical platform, performing serial dilution to assess linearity, and evaluating for macro-TSH are recommended when results are inconsistent with the clinical presentation. Failure to exclude such interference may lead to unnecessary imaging or inappropriate therapy.

#### 5.2.2. Thyroid Hormone Binding Protein Abnormalities

Disorders of thyroid hormone transport proteins should be considered, particularly familial dysalbuminemic hyperthyroxinemia. In these conditions, elevated total or even free hormone measurements may not reflect true thyrotoxicosis. Clinical euthyroidism and absence of peripheral markers of thyroid hormone excess can provide important clues.

#### 5.2.3. Assessment of Peripheral Thyroid Hormone Action

Evaluation of tissue-level thyroid hormone activity helps differentiate true hyperthyroidism from systemic resistance. Elevated SHBG and other peripheral markers support biologically active thyrotoxicosis and favor thyrotroph PitNETs over RTHβ, in which peripheral markers are often normal or only mildly increased. Measurement of the glycoprotein hormone α-subunit may provide supportive evidence in the differential diagnosis of inappropriate TSH secretion. Many thyrotroph PitNETs exhibit increased secretion of the α-subunit or an elevated α-subunit/TSH molar ratio, reflecting dysregulated hormone synthesis within tumor cells. In contrast, patients with RTHβ typically demonstrate normal α-subunit levels. However, diagnostic sensitivity is limited, particularly in microadenomas, and false-positive elevations may occur in postmenopausal women or in conditions associated with gonadotropin excess. Therefore, α-subunit measurement should be considered an adjunctive rather than definitive diagnostic tool.

#### 5.2.4. Dynamic Endocrine Testing

Thyrotroph PitNETs and RTHβ share a characteristic biochemical profile of elevated free thyroxine and triiodothyronine levels with inappropriately normal or elevated TSH concentrations, which can complicate the initial diagnostic assessment (Figure 1). However, the underlying mechanisms differ substantially. Thyrotroph PitNETs represent true hormone-producing neoplasms with autonomous TSH secretion, whereas RTHβ is a hereditary disorder caused by germline mutations in the *TRβ* gene [39], resulting in impaired tissue responsiveness to thyroid hormone without neoplastic transformation.

The T3 suppression test was historically used to distinguish thyrotroph PitNETs from RTHβ. Administration of supraphysiological doses of T3 suppresses TSH secretion in normal individuals through intact negative feedback. In patients with thyrotroph PitNETs, TSH suppression is typically blunted or absent, whereas partial suppression may be observed in RTHβ. However, diagnostic overlap exists, and sensitivity is imperfect. Furthermore, high-dose T3 administration carries potential cardiovascular risks, particularly in older patients or those with preexisting cardiac disease. With the widespread availability of high-resolution MRI and TRβ genetic testing, the T3 suppression test is now rarely performed and is generally reserved for selected, equivocal cases.

After exclusion of laboratory and binding abnormalities, dynamic testing may provide supportive evidence. The TRH stimulation test (500 μg) typically demonstrates a blunted or absent TSH response in approximately 70–80% of thyrotroph PitNETs [35,40], whereas patients with RTHβ more often exhibit a normal or exaggerated response. However, diagnostic sensitivity is variable and not absolute. In large macrotumors, rare cases of pituitary apoplexy have been reported following TRH stimulation; therefore, testing should be performed cautiously.

An alternative and potentially complementary diagnostic approach involves the use of growth hormone–releasing peptide-2 (GHRP-2), which is currently available for clinical use in Japan. GHRP-2 is widely employed for the diagnosis of severe growth hormone deficiency and is known to stimulate growth hormone and adrenocorticotropic hormone secretion, but not TSH release, in healthy individuals. Indeed, previous studies in healthy Japanese subjects demonstrated no significant change in plasma TSH levels following intravenous administration of GHRP-2 (100 μg). Preliminary data suggest that some thyrotroph PitNETs, may exhibit paradoxical TSH responses [40]. However, available evidence is limited to small series, and the role of GHRP-2 should currently be considered exploratory and hypothesis-generating pending multicenter validation.

The mechanisms underlying this paradoxical TSH response remain unclear. However, prior studies have demonstrated a positive association between growth hormone secretagogue receptor type 1a (GHSR1a) expression and in vivo responses to growth hormone–releasing peptides in corticotroph PitNETs [41]. By analogy, aberrant expression or signaling of GHSR1a in thyrotroph PitNETs may mediate GHRP-2–induced TSH secretion. Although further validation in larger cohorts is required, these findings suggest that the GHRP-2 test may serve as a novel, tumor-specific dynamic test for thyrotroph PitNETs and provide additional diagnostic information, particularly in cases where conventional testing yields equivocal results.

Octreotide LAR has been shown to be a useful and safe therapeutic treatment for thyrotroph PitNETs [42,43], and therefore SRLs in the diagnostic setting may provide additional perspective. In our previous study [40], 40% of thyrotroph PitNETs exhibit a decreased serum TSH level following octreotide stimulation (100 μg).

Accurate differentiation between thyrotroph PitNETs and RTHβ is essential, as management strategies differ fundamentally. Thyrotroph PitNETs require tumor-directed treatment, including surgery and somatostatin analog therapy, whereas RTHβ is managed conservatively without pituitary intervention. An integrated diagnostic approach incorporating dynamic testing, imaging, genetic analysis, and treatment responsiveness is therefore critical to ensure appropriate, biology-driven patient care.

#### 5.2.5. Imaging and Genetic Evaluation

Pituitary MRI should be performed once biochemical artifacts have been excluded. Identification of a pituitary tumor strongly supports the diagnosis of thyrotroph PitNETs, although incidentalomas require careful interpretation.

Short therapeutic trials with SRLs may provide additional supportive evidence, as suppression of TSH and thyroid hormone levels favors thyrotroph PitNETs. However, such response should not be used as a standalone diagnostic criterion.

Advanced imaging techniques such as 68Ga-DOTATATE PET may assist in atypical or ectopic TSH-producing tumors, expanding diagnostic capabilities beyond conventional MRI in selected cases [44].

In patients with no detectable tumor and persistent biochemical findings, genetic testing for mutations in the *TRβ* gene should be pursued to confirm RTHβ.

This structured diagnostic pathway, summarized in Figure 1 and Table 1, improves diagnostic accuracy and reduces the risk of inappropriate antithyroid drug treatment, delayed diagnosis, or unnecessary surgical intervention.

Surgical resection remains the first-line treatment in most patients, particularly in microadenomas and non-invasive macroadenomas. The recommended first-line strategies are shown according to clinical presentation, expected biochemical control rates, anticipated tumor shrinkage, predictors of response, and key practical considerations.

## 6. Therapeutic Strategies for Thyrotroph PitNETs

Management of thyrotroph PitNETs requires an individualized approach that integrates tumor size, invasiveness, biochemical activity, and patient-specific factors. Table 2 summarizes a practical, scenario-based treatment framework designed for bedside decision-making. The table outlines recommended first-line strategies according to clinical presentation, expected biochemical control rates, anticipated tumor shrinkage, predictors of response, and key practical considerations.

Surgical resection remains the first-line treatment in most patients, particularly in microtumors and non-invasive macrotumors. However, given the high prevalence of macrotumors at diagnosis, multimodal therapy is frequently required. SRLs play a central role across preoperative, adjuvant, and primary treatment settings, achieving biochemical control in approximately 70–90% of patients and tumor shrinkage in 40–60%, particularly in tumors exhibiting strong membranous SSTR2 expression.

Table 2 also highlights limitations of specific therapies, including the modest efficacy of dopamine agonists, the temporary role of antithyroid drugs, and the reserved use of radiotherapy and peptide receptor radionuclide therapy (PRRT) in refractory cases. By structuring treatment decisions according to clinical scenarios, Table 2 aims to enhance translational applicability and provide a concise reference for clinical practice.

Importantly, long-term management should integrate not only tumor control but also monitoring of systemic complications, including skeletal fragility and cardiovascular risk.

### 6.1. Surgical Management

Transsphenoidal surgery is the first-line treatment for thyrotroph PitNETs. The primary goals of surgery are complete tumor resection, normalization of TSH secretion, and relief of mass effects. Surgical remission rates vary widely, largely depending on tumor size, invasiveness, and surgical expertise. A meta-analysis of surgical outcomes reported that macrotumors constituted the majority of cases (≈79%), frequent extrasellar extension, and mixed hormone secretion profiles, emphasizing the role of multimodal therapy including surgery and SRLs [45]. Microtumors are associated with higher remission rates, whereas macrotumors frequently demonstrate cavernous sinus invasion and fibrotic consistency, making complete resection challenging [24]. Consequently, a substantial proportion of patients require additional medical therapy after surgery. Preoperative control of thyrotoxicosis is crucial to reduce perioperative cardiovascular risk [46]. In this context, SRLs are often preferred over antithyroid drugs, as they directly suppress TSH secretion and may reduce tumor volume.

### 6.2. Somatostatin Receptor Ligand Therapy

**Figure 2:**
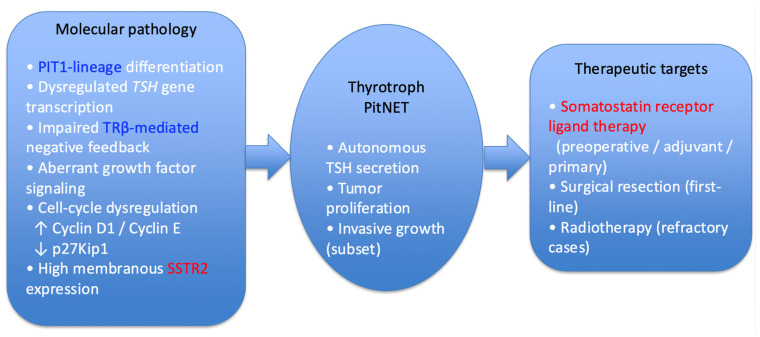
Molecular pathology and therapeutic targets of thyrotroph pituitary neuroendocrine tumors (PitNETs). Thyrotroph PitNETs are PIT1-lineage neoplasms characterized by dysregulated transcription of the thyroid-stimulating hormone (*TSH*) gene and impaired thyroid hormone-mediated negative feedback, partly due to altered thyroid hormone receptor β (TRβ) signaling. Tumor proliferation is promoted by cell-cycle dysregulation, including increased expressions of cyclin D1 and cyclin E and reduced expression of the cell-cycle inhibitor p27Kip1, together with aberrant growth factor signaling. A hallmark molecular feature is high membranous expression of somatostatin receptor subtype 2 (SSTR2), which provides a biological rationale for somatostatin analog-based therapy. Surgical resection remains first-line treatment, while somatostatin receptor ligands play a central role as preoperative, adjuvant, or primary therapy; radiotherapy is reserved for refractory cases. Receptor expression may be heterogeneous, and SSTR2 scoring methods vary among studies, which may influence predictive interpretation.

SRLs represent the cornerstone of medical treatment for thyrotroph PitNETs [47]. Thyrotroph tumors characteristically express high levels of SSTR2, which mediates potent inhibition of TSH secretion (Figure 2).

Across published clinical series, biochemical control (normalization of TSH and thyroid hormone levels) is achieved in approximately 70–90% of patients treated with long-acting SRLs, while significant tumor shrinkage is observed in approximately 40–60% of cases. Tumor reduction is more likely in patients with strong membranous SSTR2 expression on immunohistochemistry [48].

Predictors of favorable response include: (1) High SSTR2 expression intensity and membrane localization, (2) Smaller tumor size, (3) Absence of cavernous sinus invasion, (4) Lower baseline thyroid hormone levels, (5) Unlike acromegaly, MRI T2 signal intensity has not been consistently validated as a predictor of SRL responsiveness in thyrotroph PitNETs, and available evidence remains limited, and (6) Pasireotide, a multireceptor ligand with higher affinity for SSTR5, has theoretical relevance; however, given the predominance of SSTR2 expression in thyrotroph PitNETs, its role appears limited and evidence remains scarce.

### 6.3. Dopamine Agonists

Dopamine agonists, such as bromocriptine and cabergoline, have limited efficacy in the treatment of thyrotroph PitNETs. Although dopamine receptor expression has been detected in some thyrotroph PitNETs, the biochemical response is generally modest and inconsistent [6]. Dopamine agonists may be considered in selected cases, particularly in tumors co-secreting prolactin, but they are not recommended as standard monotherapy for thyrotroph PitNETs.

### 6.4. Antithyroid Drugs

Antithyroid drugs, including methimazole and propylthiouracil, may be used temporarily to control thyrotoxicosis. However, these agents do not address the underlying autonomous TSH secretion and may exacerbate pituitary TSH hypersecretion by removing thyroid hormone-mediated negative feedback [6]. Consequently, antithyroid drugs should be used cautiously and typically in combination with SRLs, rather than as standalone therapy.

### 6.5. Radiotherapy

Radiotherapy is reserved for patients with persistent or progressive disease despite surgery and medical therapy. Conventional fractionated radiotherapy and stereotactic radiosurgery have both been employed, achieving gradual tumor control over several years. However, the delayed onset of action and the risk of hypopituitarism limit the use of radiotherapy as an early treatment option [49]. Radiotherapy is therefore considered a third-line or salvage therapy in most patients with thyrotroph PitNETs.

### 6.6. Emerging and Future Therapeutic Approaches

Given the absence of recurrent driver mutations in thyrotroph PitNETs, targeted molecular therapies remain investigational. Nevertheless, advances in understanding cell-cycle dysregulation, growth factor signaling, and epigenetic mechanisms may provide novel therapeutic opportunities. Refinement of receptor-based therapies, including the development of agents with enhanced SSTR2 selectivity or combination strategies targeting multiple signaling pathways, may further improve outcomes in refractory cases.

### 6.7. Management of Complications: Skeletal and Osteometabolic Disorders

In addition to tumor-directed therapy, management of systemic complications is an essential component of the therapeutic strategy for thyrotroph PitNETs. Chronic exposure to excess thyroid hormones resulting from autonomous TSH secretion has deleterious effects on bone metabolism. Bone resorption is increased in patients with thyrotroph PitNETs compared with healthy controls, reflecting enhanced osteoclast activity driven by thyroid hormone excess [50]. Although early case reports described osteoporosis and vertebral fractures as relatively uncommon manifestations, more recent studies have demonstrated that skeletal complications are frequent and clinically relevant in this patient population. Skeletal fragility appears primarily driven by chronic thyroid hormone excess rather than intrinsic pituitary tumor effects, although cumulative endocrine disturbances may contribute in selected cases.

A particularly high prevalence of morphometric vertebral fractures has been reported in patients with thyrotroph PitNETs compared with those with nonfunctioning PitNETs [50]. Importantly, many of these fractures are asymptomatic and would remain undetected without systematic evaluation. Patients with vertebral fractures tend to be older and exhibit higher circulating free T4 levels than patients without fractures, underscoring the role of disease duration and severity of thyrotoxicosis in skeletal fragility [50]. These findings indicate that reliance on bone mineral density alone may underestimate fracture risk in thyrotroph PitNETs.

Emerging evidence suggests that treatment with SRLs may exert a protective effect on skeletal health in this setting. By directly suppressing TSH secretion and normalizing thyroid hormone levels, SRL therapy appears to reduce bone turnover and lower the risk of vertebral fractures in patients with thyrotroph PitNETs. This observation further supports the concept that SRLs serve not only as tumor- and hormone-directed therapy but also as an important component of complication management.

Based on the available evidence, a comprehensive osteometabolic evaluation should be performed in all patients with thyrotroph PitNETs at diagnosis and during follow-up. This evaluation should include assessment of bone mineral density, vertebral fracture assessment, and, when appropriate, biochemical markers of bone turnover. Early identification of skeletal involvement allows timely initiation of preventive and therapeutic interventions, including optimization of thyroid hormone control, adequate calcium and vitamin D supplementation, and anti-osteoporotic pharmacotherapy when indicated. Integrating systematic bone health management into the overall treatment algorithm is essential to reduce long-term morbidity and improve quality of life in patients with thyrotroph PitNETs.

## 7. Future Perspectives and Emerging Therapeutic Targets

Despite substantial advances in the diagnosis and management of PitNETs, several unmet needs remain, particularly in patients with invasive or refractory disease. The absence of recurrent driver mutations and the rarity of these tumors have limited the development of molecularly targeted therapies. However, recent progress in tumor classification, receptor profiling, and molecular pathology provides a framework for identifying novel therapeutic vulnerabilities and refining personalized treatment strategies.

### 7.1. Refinement of Receptor-Based Therapies

Somatostatin receptor-targeted therapy remains the most successful example of mechanism-based treatment in thyrotroph PitNETs (Figure 1). High membranous expression of SSTR2 underlies the marked efficacy of first-generation SRLs. Future therapeutic strategies may focus on optimizing receptor-based approaches through improved patient stratification, dose individualization, and development of agents with enhanced receptor selectivity or longer duration of action. Quantitative assessment of SSTR2 expression using standardized immunohistochemical scoring or functional imaging may further improve prediction of treatment response and guide personalized therapy.

Combination strategies targeting multiple receptor subtypes or signaling pathways may be explored. Although SSTR5 expression is generally lower than SSTR2 in thyrotroph PitNETs, its role in selected cases remains to be clarified. The potential utility of novel SRLs or chimeric molecules targeting both somatostatin and dopamine receptors warrants further investigation, particularly in tumors with heterogeneous receptor expression.

Given the high expression of SSTR2 in thyrotroph PitNETs, somatostatin receptor positron emission tomography imaging may provide additional diagnostic and staging information in selected cases. Although not routinely required, receptor imaging may be informative in recurrent or aggressive disease. PRRT has emerged as a conceptually aligned therapeutic option in rare aggressive, refractory, SSTR-positive pituitary tumors. Experience in thyrotroph PitNETs remains extremely limited, and current evidence is restricted to case reports and small series. Therefore, PRRT should be considered experimental and reserved for highly selected cases within specialized centers.

### 7.2. Targeting Cell-Cycle Dysregulation and Tumor Proliferation

Cell-cycle dysregulation, characterized by increased expressions of cyclin D1 and cyclin E and reduced expression of p27Kip1, represents a fundamental mechanism driving tumor growth in thyrotroph PitNETs. Therapeutic strategies aimed at restoring cell-cycle control may offer new treatment options for aggressive or treatment-resistant tumors. In this context, cyclin-dependent kinase inhibitors, which have demonstrated efficacy in other neuroendocrine and endocrine-related tumors, represent a potential area of exploration. Although clinical data in PitNETs are currently lacking, preclinical studies may help define the feasibility and safety of such approaches.

### 7.3. Growth Factor Signaling and Epigenetic Regulation

Aberrant activation of growth factor signaling pathways, including epidermal growth factor receptor and fibroblast growth factor receptor signaling, has been implicated in subsets of thyrotroph PitNETs [21,51]. Targeting these pathways may provide therapeutic benefit in selected cases, particularly when receptor expression or downstream signaling activity is demonstrated. In parallel, increasing evidence suggests that epigenetic mechanisms, such as histone modification and chromatin remodeling, contribute to dysregulated hormone gene transcription and tumor behavior [52]. Epigenetic modulators, including histone deacetylase inhibitors, may therefore represent emerging therapeutic candidates, although their clinical applicability requires careful evaluation given the benign nature of most PitNETs.

### 7.4. Advances in Molecular Diagnostics and Personalized Medicine

The integration of molecular pathology into routine diagnostic workflows represents a major advance in the management of thyrotroph PitNETs. Lineage-based classification using PIT1 IHC, combined with assessment of hormone expression and somatostatin receptor profiling, provides a robust framework for diagnosis and therapeutic decision-making. Future diagnostic strategies may incorporate multi-omics approaches, including transcriptomic and epigenomic profiling, to further refine tumor classification, identify prognostic markers, and uncover novel therapeutic targets. Such approaches may be particularly valuable in rare or atypical cases where standard treatments are ineffective.

### 7.5. Overcoming Challenges Related to Rarity and Clinical Evidence

The extreme rarity of thyrotroph PitNETs remains a major obstacle to clinical research. Prospective randomized trials are unlikely to be feasible, underscoring the importance of international collaboration, multicenter registries, and standardized data collection. Harmonization of diagnostic criteria, treatment protocols, and outcome measures will be essential to generate high-quality evidence and improve comparability across studies. In this context, comprehensive reviews that integrate biological rationale with accumulated clinical experience play a critical role in guiding practice and informing future research directions.

### 7.6. Toward a Tumor-Oriented Therapeutic Paradigm

Collectively, these emerging perspectives support a shift toward a tumor-oriented therapeutic paradigm for thyrotroph PitNETs, in which treatment decisions are guided by tumor biology rather than solely by biochemical abnormalities. Receptor expression, lineage identity, and molecular features should be incorporated into individualized treatment algorithms. Continued advances in molecular understanding and therapeutic innovation hold promise for improving outcomes in patients with refractory or aggressive disease, while minimizing treatment-related morbidity.

## 8. Conclusions

Thyrotroph PitNETs are rare PIT1-lineage PitNETs characterized by autonomous TSH secretion and impaired thyroid hormone-mediated negative feedback. Although transsphenoidal surgery remains the first-line treatment, complete surgical remission is often difficult to achieve due to tumor size and invasiveness. A defining molecular feature of thyrotroph PitNETs is their high expression of SSTR2, which provides a strong biological rationale for SRL-based therapy. SRLs play a central role across the therapeutic spectrum, including preoperative preparation, postoperative adjuvant treatment, and primary therapy in selected patients. Accurate differentiation from RTHβ is essential to avoid inappropriate surgical or medical interventions.

Advances in molecular pathology, particularly lineage-based classification and receptor profiling, have refined diagnostic accuracy and enabled personalized treatment strategies, ultimately improving clinical outcomes for patients with thyrotroph PitNETs. These data support a receptor-based, tumor-oriented therapeutic paradigm that may be extended by future molecularly targeted strategies. In addition to tumor control and normalization of thyroid hormone excess, systematic assessment and management of skeletal complications, particularly vertebral fractures, should be integrated into the therapeutic strategy for thyrotroph PitNETs to reduce long-term morbidity. Ultimately, recognition of thyrotroph PitNETs as PIT1-lineage neoplasms with receptor-defined therapeutic vulnerabilities underscores the importance of integrating molecular classification with precision endocrine oncology.

## Figures and Tables

**Table 1:** Differential diagnosis between thyrotroph PitNETs and resistance to thyroid hormone β (RTHβ). Key clinical, biochemical, radiological, and molecular features distinguishing thyrotroph pituitary neuroendocrine tumors (PitNETs) from RTHβ.

Diagnostic Step	Feature	Thyrotroph PitNETs	RTHβ
	Disease entity	Functional pituitary neuroendocrine tumor	Hereditary thyroid hormone action disorder
	2022 WHO classification	Thyrotroph PitNET (PIT1-lineage tumor)	Not classified as a PitNET
	Pathogenesis	Autonomous TSH secretion from neoplastic thyrotrophs	Germline TRβ mutation causing tissue resistance
	Inheritance	Sporadic	Autosomal dominant (rarely sporadic)
	Family history	Usually absent	Often positive
1	Assay interference	None	Heterophile antibodies, biotin effect
2	Binding disorders	Normal	Familial dysalbuminemic hyperthyroxinemia (FDH)
3	Peripheral markers of thyroid hormone action	Elevated sex hormone–binding globulin (SHBG)	Normal or mildly elevated SHBG
3	α-Subunit of glycoprotein hormones	Often elevated	Normal
	TSH regulation	Autonomous, partially resistant to thyroid hormone feedback	Inappropriately regulated but non-autonomous
4	TRH stimulation test	Blunted or absent TSH response	Preserved or exaggerated TSH response
4	Response to somatostatin receptor ligands (SRLs)	Marked TSH and thyroid hormone suppression	Minimal or no response
4 (optional)	T3 suppression test	Incomplete or absent suppression	Partial suppression possible
5	Pituitary MRI	Discrete pituitary tumor (often macroadenoma)	Normal pituitary or incidental findings
6	Genetic background	Somatic alterations (no recurrent driver mutation identified)	Germline TRβ mutations
	Somatostatin receptor expression	High SSTR2, moderate SSTR5	No pathological overexpression
	Tumor shrinkage with SRLs	Frequently observed	Not applicable
	Primary treatment	Transsphenoidal surgery ± SRLs	Conservative management
	Role of SRLs	Preoperative, adjuvant, or primary therapy	Not indicated
	Prognosis	Depends on tumor size and invasiveness	Generally benign, lifelong condition

**Table 2:** Practical Treatment Algorithm for Thyrotroph Pituitary Neuroendocrine Tumors.

Clinical Scenario	Recommended First Step	Expected Biochemical Control	Tumor Shrinkage	Key Predictors of Response	Practical Considerations
Microtumor without invasion	Transsphenoidal surgery	70–90%	Immediate resection	Small size	High remission likelihood
Macrotumor (non-invasive)	Surgery ± preoperative somatostatin receptor ligands (SRLs)	60–80%	40–60% with SRLs	Strong membranous SSTR2	Preoperative euthyroidism recommended
Invasive macrotumor	SRLs as primary or adjuvant therapy	70–90%	40–60%	SSTR2 intensity	Often requires multimodal therapy
Residual tumor post-surgery	SRLs	70–90%	40–60%	SSTR2 expression	Long-term monitoring required
Co-secreting PRL tumor	SRLs ± dopamine agonist	Variable	Limited	Dopamine receptor expression	Dopamine agonist rarely sufficient alone
Persistent hyperthyroidism awaiting surgery	SRLs (preferred)	Rapid control	Possible shrinkage	—	Avoid antithyroid monotherapy
Severe thyrotoxicosis	SRLs ± short-term antithyroid drugs (ATDs)	Symptomatic control	None (ATD)	—	ATDs may increase TSH
Refractory/aggressive tumor	SRLs → Radiotherapy → PRRT (selected cases)	Gradual	Stabilization	High SSTR expression	Specialized centers only

## Data Availability

Data are contained within the article.
